# Provider imposed restrictions to clients’ access to family planning in urban Uttar Pradesh, India: a mixed methods study

**DOI:** 10.1186/1472-6963-13-532

**Published:** 2013-12-23

**Authors:** Lisa M Calhoun, Ilene S Speizer, Rajiv Rimal, Pooja Sripad, Nilesh Chatterjee, Pranita Achyut, Priya Nanda

**Affiliations:** 1 Carolina Population Center, University of North Carolina at Chapel Hill, Chapel Hill, USA; 2 Department of Maternal and Child Health, Gillings School of Global Public Health, The University of North Carolina at Chapel Hill, Chapel Hill, NC, USA; 3 Department of Prevention and Community Health, George Washington University, Washington, DC, USA; 4 Department of International Health, Johns Hopkins University, Baltimore, USA; 5 Center for Communication Programs, Johns Hopkins University, Lucknow, India; 6 International Center for Research on Women, Delhi, India

**Keywords:** Provider barriers, Family planning, Eligibility barriers, Uttar Pradesh, Urban, India

## Abstract

**Background:**

Medical barriers refer to unnecessary policies or procedures imposed by health care providers that are not necessarily medically advised; these restrictions impede clients’ access to family planning (FP). This mixed methods study investigates provider imposed barriers to provision of FP using recent quantitative and qualitative data from urban Uttar Pradesh, India.

**Methods:**

Baseline quantitative data were collected in six cities in Uttar Pradesh, India from service delivery points (SDP), using facility audits, exit interviews, and provider surveys; for this study, the focus is on the provider surveys. More than 250 providers were surveyed in each city. Providers were asked about the FP methods they provide, and if they restrict clients’ access to each method based on age, parity, partner consent, or marital status. For the qualitative research, we conducted one-on-one interviews with 21 service providers in four of the six cities in Uttar Pradesh. Each interview lasted approximately 45 minutes.

**Results:**

The quantitative findings show that providers restrict clients’ access to spacing and long-acting and permanent methods of FP based on age, parity, partner consent and marital status. Qualitative findings reinforce that providers, at times, make judgments about their clients’ education, FP needs and ability to understand FP options thereby imposing unnecessary barriers to FP methods.

**Conclusions:**

Provider restrictions on FP methods are common in these urban Uttar Pradesh sites. This means that women who are young, unmarried, have few or no children, do not have the support of their partner, or are less educated may not be able to access or use FP or their preferred method. These findings highlight the need for in-service training for staff, with a focus on reviewing current guidelines and eligibility criteria for provision of methods.

## Background

Understanding clients’ access to family planning (FP) methods extends beyond individual or household characteristics, contraceptive security, physical accessibility and economic issues, to also include restrictions or assumptions about the client that a service provider may impose on the provision of these methods [1-3]. Unnecessary restrictions have many implications for women, including unintended pregnancy and unsafe abortion, as well as affecting both clients’ access to FP and the quality of care they receive when seeking FP services [1,3].

Medical barriers refer to unnecessary policies or procedures imposed by medical providers that are not necessarily medically advised and result in providers impeding clients’ access to FP [2,4-6]. Shelton et al. (1992) identified 6 types of medical barriers for FP provision: contraindications, eligibility, process hurdles, type of provider requirements, provider bias, and regulation [2]. These barriers arise for a number of reasons, and are not necessarily a result of negative intentions on the part of the provider [2,5,7]. Rather, service delivery guidelines may not be up-to-date, or may not be followed by providers. Further, providers may require exams or tests that are not needed, or be unwilling to prescribe a method due to misinformation about the association between a health condition and use of a method, such as concerns about contraindications between diabetes and oral contraceptive pills.

This study focuses on eligibility barriers, which are a result of a provider deciding which FP methods to offer on the basis of his/her own cultural and social norms or on the basis of his/her observations about a client’s personal characteristics, such as religion, caste, education, age, parity and marital status [2,8]. These types of barriers have been explored previously through quantitative and qualitative studies [4,9-12]. Prior research from sub-Saharan Africa has shown that providers limit clients’ access to FP based on marital status, age and parity of the clients [4,5,12]. When interacting with adolescent and young adult clients (e.g., under age 25 years), some providers have personal opinions or morals regarding sexual activity among youth and unmarried young people and therefore inappropriately limit these young people’s access to reproductive health services [4,13]. This can result in young people having limited access to both FP methods [4] and information or counseling about FP [14]. These studies from sub-Saharan Africa show that providers’ personal morals can impede clients’ access to family planning services.

To date, less is known about provider barriers in the Asian context. One study from India showed that, because providers tend to serve a high volume of clients, they may not have adequate time to counsel clients about the full range of methods available to them [15]; this reflects poor quality of services but is not an eligibility barrier. In Uttar Pradesh (UP), the focus site of this study, a 1995 study using the PERFORM data from rural UP, indicated inconsistencies between perceptions of FP availability at the community level and actual supply-side characteristics of the facilities such as staff gender composition, provider training or re-training within the last three years, whether the provider could provide the correct advice to clients regarding missed pills, and range of methods available [16]. The PERFORM study indicated that perceived availability did not necessarily correspond to actual facility characteristics; of note, the supply-side characteristics that were explored were not necessarily eligibility barriers [16].

Schuler and colleagues’ (1985) research in Nepal found that providers made judgments about clients’ intelligence and ability to use specific methods effectively [17]. Further, they found from both the provider and client perspectives that the biases were greatest toward poor, low-caste clients and that clients with only one or two children were often denied sterilization [17]. These previous studies highlight the importance of examining provider-imposed barriers to FP use in a variety of contexts, as provider barriers likely vary considerably by country, region, and culture. To the authors’ knowledge, this current study is the only study that specifically addresses provider barriers in urban UP.

Generally, urban areas in India have better access to and greater availability of health care through both public and private sector facilities as compared to rural areas [18]. Despite greater access in urban areas, access to health care is still somewhat limited, as only 41.5% of urban women reported any contact with a healthcare provider in the three months prior to the 2005/2006 National Family Health Survey (NFHS) [19].

Recent efforts have been made by the Government of India (GoI) to improve access and provide effective health care across India. As an example, the National Rural Health Mission (NRHM) was rolled out in 2005 and a similar framework was laid out for the National Urban Health Mission (NUHM), which received official approval in 2013. The NUHM focuses on the primary health care needs of the urban poor through activities such as revitalization of the existing system, community outreach, and partnerships with the private sector and health insurance schemes [20]. The focus on urban areas by NUHM will help ensure availability of primary health facilities in these urban sites, and aims to improve utilization levels and referral systems especially for urban slum dwellers [20].

This mixed methods study fills a void by using quantitative and qualitative data to investigate provider imposed barriers in an urban context using recently collected data from cities in Uttar Pradesh, India. Expanding the knowledge base on this topic in urban UP will help elucidate and understand barriers to FP use, and provide the basis for designing programs to reduce identified barriers and increase access to FP in urban UP.

## Methods

### Context

In 2009, the Measurement, Learning & Evaluation Project (MLE) was initiated to evaluate the Urban Reproductive Health Initiative (URHI) programs, which focus on improving FP and reproductive health services of the urban poor in Kenya, Nigeria, Senegal and India. In India, the project is called the Urban Health Initiative (UHI) and is led by FHI 360. UHI is working in 11 major urban cities in the state of Uttar Pradesh (UP).

UP, with an estimated population of 199 million in 2011, is the most populous state in all of India [21]. UP is also one of the least developed states in terms of the percentage of households with electricity, female literacy, and infant mortality [19]. Further, nearly one quarter of UP’s population resides in urban areas [21] and UP has the greatest number of urban residents in a single state living below the poverty line [22].

Previously published data from the MLE study in UP shows that health service needs are high. In terms of reproductive health and FP measures, the total fertility rate ranged from 2.84 to 4.03 children per woman across the six study cities: Agra, Aligarh, Allahabad, Gorakhpur, Moradabad and Varanasi [23]. Modern FP use among married women of reproductive age ranged from 38-54%, with the majority of women relying on sterilization (34-54%) and condoms (31-54%) [23,24].

MLE data also showed that public facilities were a major source of FP services, with between 28% and 40% of modern FP users citing a public sector facility as their source [23]. The public sector was the most frequently mentioned source of female sterilization, whereas women most frequently cited the pharmacy or their husbands as their source for condoms [23]. In terms of FP methods, across all study cities, more than half of high volume public and private facilities (50-100%) provided 4 or more contraceptive methods, though there was considerable variation across the cities in the number of methods provided at smaller public and private facilities, with those offering four or more methods ranging from 0 to 48% [23].

This mixed methods study uses quantitative data from service delivery points from the six cities (Agra, Aligarh, Allahabad, Gorakhpur, Moradabad, Varanasi) in the MLE project and qualitative data from health providers collected in Agra, Aligarh, Allahabad and Gorakhpur as part of the of baseline formative research.

### Quantitative methods

Baseline quantitative data for the MLE project in India was collected between January-July, 2010 and included a household survey and a survey of service delivery points (SDP) in the study cities. For the household survey, a representative sample of about 3,000 women and 1,500 men was selected from slum and non-slum primary sampling units in each of the six study cities. The methods for the household survey that is not the focus of the analysis for this study have been described previously [23,24].

A survey of service delivery points (SDP) in the six study cities included facility audits, exit interviews, and provider surveys, as well as a survey of pharmacies. The sample of facilities includes all public hospitals, all public health centers, all private hospitals, and a selection of private clinics and nursing homes from each city. Private clinics/doctors and pharmacies in each city were selected based on those mentioned by women in the household survey; at the primary sampling unit level, women’s responses were tallied and the most frequently mentioned private facilities and pharmacies were selected for survey. Client exit surveys were carried out in high volume public and private hospitals and nursing homes. Providers were surveyed at all facility types. All clients and providers gave verbal consent to participate in this study. This study focuses on the data from the provider and pharmacy surveys.

The facility audit was administered to the facility administrator to gather information on all types of staff providing health services, including their position, sex, full-time work status, and whether they provided maternal health, child health and FP services. Lists of providers who were providing family planning or maternal and child health services, including gynecologists, pediatricians, Ayush doctors^a^, nurses, midwives, traditional birth attendants (TBA)^b^, and other staff formed the sampling frame for their selection in the study. From each category of provider, a maximum of two providers were selected randomly for the survey. However, the actual number of staff of each cadre varied due to their availability and interest to participate. A total of 1,752 providers were surveyed from 732 facilities, ranging from 226 to 411 providers in each study city.

The provider survey included questions on training, provision of FP methods, integration of FP with other services, and availability of methods at the facility. Providers were asked about the FP methods they were sufficiently knowledgeable about to provide, and whether they restricted clients’ access to each method based on age, parity, partner consent, or marital status.

The pharmacy survey was administered to the pharmacist, manager/facility administrator, or infrequently another staff member, at the pharmacy at the time of survey. A total of 517 pharmacies were included across the six cities, with one person (pharmacist, manager/administrator, or other person) surveyed per site. This survey included questions on training, barriers to provision of methods, availability of methods, brands available, and storage and stocking conditions.

This study primarily explored eligibility barriers. A minimum age barrier for the intrauterine contraceptive device (IUCD), male condoms, oral contraceptive pills (OCP), and injectables was defined if the provider required the client to have a minimum age of 18 to receive these FP methods. This definition did not consider it a barrier if a provider restricted access to a method for women who were under 18; this conservative definition was based on the legal age at marriage in India (18 years of age). A maximum age barrier for the IUCD, condoms, OCP, and injectables was defined if the provider restricted access to these methods for clients age 48 and younger. Therefore, it was not considered a maximum age barrier if providers said they restricted methods to women who were 49 years old or older, as these women likely were menopausal and not in need of family planning. The definition of a parity barrier was that the provider required that a client have any number of children before supplying a method. Parity barriers were examined by the minimum number of children required. Barriers for partner consent and marital status were defined if providers answered positively that they restricted access to FP if the woman does not have her partner’s consent or is not married.

Table 1 summarizes Government of India (GoI) and other guidelines for provision of FP methods in India. The table includes the name of the guidelines, requirements for method provision given within the guidelines, which providers are allowed to provide each method, and if the method is part of the GoI package of FP methods. For female sterilization, the GoI guidelines stipulate that clients should be ever-married, between the ages of 22 and 49, have at least one child whose age is above one year unless the sterilization is medically indicated, have not undergone sterilization in the past (not applicable in cases of failure of previous sterilization), be in a sound state of mind so as to understand the full implications of sterilization, and certified by a psychiatrist if mentally ill (which further required a statement from the legal guardian/spouse regarding the soundness of the client’s state of mind) [25]. Based on these GoI guidelines, providers were considered to have an age barrier if they restricted access to a client between 23 and 49 years of age. Based on the GoI guidelines, only doctors can provide female sterilization [25].

**Table 1:** Guidelines on FP method provision in India

**Method**	**Guidelines on eligibility barriers**	**Guidelines on which providers allowed to provide method**	**Included in GoI FP method list**	**Name of guideline(s)**
Female sterilization	**Age:** 22-49 years	Doctors	Yes	Division of Research Studies and Standards, Ministry of Health and Family Welfare, Government of India: Standards for Female and Male Sterilization Services [25]
	**Parity:** 1+, above 1 year of age			
	**Marital status:** Ever-married			
IUCD	**Age:** Nursing staff needs to refer women with age <20 to doctor for IUCD insertion	Doctors	Yes	Family Planning Division, Ministry of Health and Family Welfare, Government of India: IUCD manual for nursing personal [27]
	**Parity:** Nursing staff needs to refer nulliparous women to doctor for IUCD insertion	Nurses		Family Planning Division, Ministry of Health and Family Welfare, Government of India: IUCD Reference Manual for Medical Officers [26]
		Lady Health Visitors		
		Auxiliary Nurse Midwives (ANMs)		
Injectables	**Age:** menarche to < 40 years - Use method in any circumstance; ≥ 40 years – Generally use the method	Doctors**	No	USAID/India Dimpa Initiative [28]
	**Parity:** Use method in any circumstance for any parity			WHO medical eligibility criteria for contraceptive use, 2009 [29]
Pill	None	Doctors	Yes	Family Planning Division, Ministry of Health and Family Welfare, Government of India: List of drugs being provided in ASHA kit [30]
		Nurses		
		Midwives		
		TBAs/CHW*		
		Pharmacies		
Condom	None	Doctors	Yes	Family Planning Division, Ministry of Health and Family Welfare, Government of India: List of drugs being provided in ASHA kit [30]
		Nurses		
		Midwives		
		TBAs/CHW*		
		Pharmacies		

*Condoms and oral contraceptive pills are included in the National Rural Health Mission Accredited Social Health Activist (ASHA, a community health worker) kit. ASHAs are the base of the health provider system in India.

**Injectable contraceptives are primarily available through the Dimpa network in India, and within this network, only high level providers can give injections.

The GoI has guidelines for IUCD use, and guidelines indicate that doctors and nursing staff can insert the IUCD [26,27]. The GoI guidelines do not restrict the use of the IUCD based on age, parity, marital status or partner consent; however, the GoI requires nurses to refer nulliparous and clients under 20 years of age to doctors for IUCD insertion [26,27].

The GoI does not have specific guidelines for injectable use, as injectables are not part of the GoI package of FP methods. The Dimpa network serves as the primary provider of injectables in the private sector, and works to build the capacity of private doctors and enroll them in a network of clinics to increase knowledge of and access to injectables [28]. The Dimpa network follows the WHO medical eligibility criteria for provision of injectables, which recommends that clients from menarche to <40 years of age and women of any parity not be restricted from using injectables and that women 40 or older generally should be allowed to use the method with clinical judgment [29].

The National Rural Health Mission indicates that community health workers, the base of the health provider system, are able to provide both condoms and pills [30]. The available guidelines do not stipulate any specific guidelines related to age, parity, marital status or partner consent [30].

### Qualitative methods

In Agra, Aligarh, Allahabad and Gorakhpur, we conducted formative research for which we collected data from focus groups (among women, their husbands, and their mothers-in-law) and in-depth interviews (with health professionals and service providers). In this paper, we focus only on the in-depth interviews conducted with service providers. Providers included in this study were identified through our on-site program implementers with extensive knowledge about the areas. We sought to balance providers from government and private clinics and focused on those who tended to work in relatively high-volume areas.

Interviewers underwent a weeklong training session that focused on human subjects’ issues, survey tools and interviewing skills. As part of the training, mock interviews were conducted and interviewers were provided feedback to improve their skills.

We conducted one-on-one interviews with 21 service providers, including doctors, anganwadi workers^c^ and frontline health workers (or service promoters^d^), using a semi-structured interview guide. Interviews were conducted in the service providers’ office or in a convenient location for the provider. Each in-depth interview lasted approximately 45 minutes. Interviews were tape recorded, subsequently transcribed and then translated into English (for interviews done in Hindi). The translated text was coded deductively, based on the identified thematic areas from the quantitative arm, using Atlas.ti software. Ambiguities in meaning were resolved by referencing original Hindi transcripts.

This project was approved by the Futures Group India Institutional Review Board and Institutional Review Boards at The University of North Carolina at Chapel Hill and the International Center for Research on Women.

## Results

### Quantitative results

Key characteristics of the 1,752 facility-based health providers available for this analysis are presented in Table 2. About 34% of surveyed providers were doctors, 14% Ayush doctors, 25% nurses, 6% midwives, 10% traditional birth attendants (TBA), and 11% other staff. The majority of providers in our sample were from private facilities; this reflects the fact that the facility survey included a large number of small, private clinics.

Overall, less than 25% of health facility providers reported receiving any in-service training on family planning, and of those who did, only about 16% of doctors and midwives, 25% of nurses, and 0% of TBAs received this in-service training within the last year. Over 50% of doctors who provided sterilization had not ever attended an in-service training on this topic (data not shown). Notably, having ever received in-service training on clinical skills for the IUCD was reported by nearly 75% of doctors and nurses (data not shown).

Pharmacy audits were carried out in 70-110 pharmacies per city, for a total of 517 pharmacies (see Table 3). Of the staff members surveyed, about 91% were managers/facility administrators, 8% pharmacists, and 1% other staff members. Although 96% of staff from pharmacies ever provide or counsel on family planning, only about 28% of these persons reported having any training on FP service delivery (Table 3).

**Table 2:** Background characteristics of providers surveyed from health facilities

** Characteristic**	**Provider type**						
	**Doctors**	**Ayush doctors**	**Nurses**	**Midwives**	**Traditional birth attendants**	Other*	**Total**
	**n = 598**	**n = 260**	**n = 441**	**n = 95**	**n = 172**	**n = 186**	**n = 1752**
Facility type provider working in							
High volume public	8.4	1.2	7.3	15.8	11.1	13.4	8.2
High volume private	25.8	4.6	29.9	16.8	40.7	11.3	23.1
Public	5.9	3.5	6.6	53.7	21.5	54.3	15.0
Private	60.0	90.8	56.2	13.7	26.7	21.0	53.7
Number of years at facility							
Less than one year	5.2	3.5	13.8	11.6	5.8	8.1	7.8
1-2 years	9.5	10.4	20.6	25.3	22.7	18.3	15.5
3-5 years	17.6	15.8	29.3	16.8	23.3	22.0	21.2
6-9 years	18.2	17.3	13.2	13.7	19.8	15.1	16.4
10-19 years	28.6	29.2	15.2	14.7	18.0	21.0	22.7
20+ years	20.4	23.9	7.5	16.8	9.9	14.0	15.8
Don’t remember/missing	0.5	0.0	0.5	1.1	0.6	1.6	0.6
Sex of respondent							
Male	48.0	90.4	12.5	2.1	1.7	33.9	36.8
Female	52.0	9.6	87.5	97.9	98.3	66.1	63.2
Received any in-service training on FP							
Yes	23.9	25.4	14.3	48.4	4.7	52.7	24.2
No	76.1	74.6	85.7	51.6	95.4	47.3	75.8
Received any in-service training on FP in the last year	n = 143	n = 66	n = 63	n = 46	n = 8	n = 98	n = 424
Yes	15.4	24.2	25.4	17.4	0.0	13.3	17.7
No	72.7	68.2	66.7	78.3	75.0	74.5	72.2
Don’t remember/missing	11.9	7.6	7.9	4.4	25.0	12.2	10.1
Total	100.0	100.0	100.0	100.0	100.0	100.0	100.0

*Other includes health educators, social workers, lady health volunteers and administrators.

**Table 3:** Background characteristics of pharmacy providers surveyed at pharmacies

**Characteristic**	**n = 517**
Person surveyed	
Pharmacist	8.1
Manager/Administrator	91.1
Other	0.8
Sex of respondent	
Male	99.4
Female	0.6
Pharmacy has a trained pharmacist employed there	
Yes	83.0
No	17.0
Staff from pharmacy ever provide information and counseling related to FP to clients	
Yes	96.3
No	3.5
Missing	0.2
Ever received any training on FP	
Yes	28.2
No	71.6
Don’t know	0.2
Total	100.0

Table 4 presents the number of family planning providers who provide the various methods. The table combines providers from all six cities and is organized by type of provider and eligibility restriction: age restrictions (a minimum age or a maximum age), partner consent restrictions, and marital status restrictions. Because minimal differences in the restrictions were observed across facility type, all types of facilities were combined. Notably, the number of doctors who provided each method was higher than the number of nurses and midwives. Few TBAs provided the methods of interest to this analysis.

**Table 4:** Number of family planning providers who provide each method and who restrict clients’ eligibility to use a method for reasons of age, marital status or partner’s consent, by method, according to type of provider in Uttar Pradesh

**Restriction/method**	**Doctor**		**Nurse**		**Midwife**		**TBA**	
	**Number**	**Percent**	**Number**	**Percent**	**Number**	**Percent**	**Number**	**Percent**
Minimum Age								
Pill	308	31.5	64	62.5	71	42.3	13	46.2
Condom	281	12.8	66	10.6	70	17.1	14	7.1
IUCD	283	70.3	90	78.9	69	75.4	19	63.2
Injectables	219	48.9	NA	NA	NA	NA	NA	NA
Sterilization	213	70.9	NA	NA	NA	NA	NA	NA
Maximum Age								
Pill	308	79.9	64	95.3	71	98.6	13	84.6
Condom	281	5.7	66	25.8	69	8.7	14	28.6
IUCD	284	89.1	90	85.6	69	97.1	19	84.2
Injectables	219	80.4	NA	NA	NA	NA	NA	NA
Sterilization	212	81.1	NA	NA	NA	NA	NA	NA
Partner’s consent								
Pill	307	20.5	66	50.0	72	27.8	13	46.2
Condom	280	13.2	67	29.9	71	22.5	15	13.3
IUCD	284	69.7	96	85.4	70	72.9	21	47.6
Injectables	217	34.1	NA	NA	NA	NA	NA	NA
Sterilization	214	90.7	NA	NA	NA	NA	NA	NA
Marital status								
Pill	307	47.6	66	77.3	72	72.2	13	61.5
Condom	281	28.8	67	50.8	71	52.1	15	20.0
IUCD	284	97.5	96	89.6	70	98.6	21	95.2
Injectables	217	68.2	NA	NA	NA	NA	NA	NA
Sterilization	214	98.6	NA	NA	NA	NA	NA	NA

Note: Number of providers differs by barrier due to nonresponse or missing data for these questions; NA - Not Applicable.

Approximately 30% of doctors reported that they restricted eligibility to pills based on a minimum age, whereas more than 70% restricted access to sterilization and IUCD based on a minimum age requirement. Nurses and midwives exceeded 70% for restriction of the IUCD based on a minimum age. Even though condoms are a barrier method with no hormonal side effects, minimum age restrictions for condoms were imposed by 12% of doctors and midwives restricted their provision most frequently (17%). Approximately 50% of doctors said they restrict access to injectables based on a minimum age, though injectables are not commonly available or used in Uttar Pradesh [28]. The average minimum age for provision of pills, condoms and IUCD was between 19 to 22, while for sterilization it was 26 years of age (data not shown).

Providers report more frequently restricting clients’ access to methods based on a maximum age than a minimum age. Approximately 80% of doctors restricted a clients’ access to pills, IUCD, sterilization and injectables based on clients’ maximum age; they restricted women 48 and younger access to these methods, even though some women in this age range were likely to need a family planning method. Nurses, midwives and TBAs all frequently reported restricting clients’ access to pills and IUCD based on a maximum age, whereas a lower percent of these providers restricted access to condoms for clients based on a maximum age. On average, the maximum age reported for provision of pills, sterilization, and the IUCD was 40 years of age (data not shown). The average maximum age reported for provision of condoms was 49 years, though it ranged from 35 to 60 years, suggesting that providers were less restrictive for older women using condoms (data not shown); this may be a reflection of providers’ focusing on the disease prevention function of condoms.

Doctors reported restricting clients’ access to long acting and permanent methods such as sterilization (91%) and IUCD (70%) based on the need for partner consent more frequently than they restricted short term methods, such as condoms (13%) or pills (21%). Nurses also reported restricting clients’ access to pills (50%), condoms (30%) and IUCD (85%) based on partners’ consent. Approximately one quarter of midwives restricted client access to pills and condoms based on partner consent and nearly 75% restricted access to the IUCD based on partner consent. Approximately half of TBAs restricted access to pills and IUCD based on partner consent, while only 13% restricted access to condoms based on partner consent.

Restrictions for sterilization and IUCD based on marital status were common amongst all provider types. Nearly 99% of doctors restricted access to sterilization based on marital status, which may be related to GoI guidelines requiring women to be ever-married. Doctors less frequently restricted access to pills (48%), condoms (29%) and injectables (68%) based on marital status, though these percentages were relatively high as compared to the other restriction types, especially for condoms. About 50% of nurses and midwives and only 20% of TBAs restricted a client’s access to condoms based on marital status. Pill restrictions based on marital status were also common, at 77% of nurses, 72% of midwives, and 62% of TBAs.

Table 5 presents the number of pharmacy providers who provide each method and responded to the restriction questions. Only condoms and pills are presented as these were the two main methods available in pharmacies. Approximately 30% of pharmacy providers restricted access to pills and 3% restricted access to condoms based on a minimum age. Based on maximum age, restrictions for pills were about 47% and for condoms at about 2%. For both partner consent and marital status, restrictions for pills and condoms were relatively low, ranging from 3% to 11%. Overall, pharmacy providers had relatively low levels of restrictions to access for condoms and pills.

**Table 5:** Number of pharmacy providers who provide each method and who restrict clients’ eligibility to use a method for reasons of age, marital status or partner consent, by method in Uttar Pradesh

**Restriction/method**	**Pharmacy provider**	
	**Number**	**Percent**
Minimum Age		
Pill	494	29.8
Condom	506	3.4
Maximum Age		
Pill	494	46.8
Condom	506	2.2
Partner consent		
Pill	500	11.0
Condom	506	8.9
Marital status		
Pill	500	4.2
Condom	506	2.6

Note: Number of providers differs by barrier due to nonresponse or missing data for these questions.

Table 6 presents providers’ self-reported restriction to family planning based on a woman’s number of children (parity). Approximately 90% of providers restricted access to female sterilization and IUCD based the client’s parity. Of the doctors who restricted access to the IUCD based on parity, 65% required the client to have one child. In contrast, of the TBAs who restricted access to the IUCD based on parity, 63% required that the client have two children. Restriction to female sterilization based on parity was nearly universal amongst doctors, as dictated by the GoI guidelines, though of those who restrict, 83% required a client to have at least two children, which is in contradiction to the one child requirement stipulated in the GoI guidelines [25]. Parity restrictions were imposed for pills by 66% of nurses, while only 20% of doctors and 25% of TBAs restricted access to pills due to parity. Of those providers who reported restricting a client’s access to pills based on parity, nearly 50% required a client to have two children to be provided pills. Fewer providers (around 10%) restricted provision of condoms to clients by parity, with midwives most frequently restricting access at 17%.

**Table 6:** Number of family planning providers who provide each method and who restrict clients’ eligibility to use a method for reasons of parity, by method, according to type of provider in Uttar Pradesh

**Method**	**Number of providers that provide method**	**Percent of providers that restrict method based on parity**	**Number of providers who restrict**	**Of the providers that restrict, percent that report the minimum number of children a client must have**		
				**One child**	**Two child**	**Three or more children**
**Pill**						
Doctor	306	19.6	60	48.3	50.0	1.7
Nurse	64	65.6	42	42.9	57.1	0.0
Midwife	71	32.4	23	56.5	43.5	0.0
TBA	13	15.4	2	50.0	50.0	0.0
Pharmacy	497	2.2	11	45.5	45.5	9.1
**Condom**						
Doctor	281	6.8	19	31.6	68.4	0.0
Nurse	64	12.5	8	25.0	62.5	12.5
Midwife	71	16.9	12	33.3	66.7	0.0
TBA	14	7.1	1	0.0	100.0	0.0
Pharmacy	505	0.4	2	0.0	100.0	0.0
**IUCD**						
Doctor	284	87.0	247	64.6	32.5	2.8
Nurse	93	91.4	85	52.9	47.1	0.0
Midwife	69	89.9	62	69.4	27.4	3.2
TBA	18	88.9	16	37.5	62.5	0.0
**Injectables**						
Doctor	220	41.8	92	73.9	26.1	0.0
**Sterilization**						
Doctor	213	94.4	201	10.5	83.1	6.5

### Qualitative results

In-depth interviews with service providers about their roles and dialogues with women provided a more contextualized understanding of restrictions imposed on FP use in these urban settings. The qualitative results from service provider interviews fall broadly into three over-arching themes, “service providers as guides, not decision-makers”, “gender relations and FP access” and “perceptions of socioeconomic and normative influence on FP knowledge and access.”

#### Service providers as guides, not decision-makers

Similar to the quantitative findings, qualitative data suggest fewer restrictions around condoms and pills compared to more invasive family planning methods, with little difference between provider types. Age and parity restrictions and risk associated with each FP method were frequently discussed as markers of what types of FP methods were offered and thought to be appropriate for women and couples. Provider perceptions of the basic education and health literacy of a patient were reported to influence whether or not they restrict access to or encourage certain FP methods over others. Despite sharing their perspectives and opinions through conversation with clients seeking FP services, providers emphasized their role as a guide and not a decision-maker.

Service providers were often emphatic in qualitative interviews that they catered to the needs of their clients. Many of the providers repeatedly justified their FP recommendations based on conversations they had with clients, and they characterized their style as “client driven”:

*“It depends how they converse with us. Like if they want guidance about family planning, directly then I tell them. If they have some misconceptions, then I try to clarify. Then we conclude what they need to use. Then final decisions are theirs, in terms of what they choose.”* (Service promoter, Agra)

As guides, service providers often discussed misconceptions and potential side-effects around FP methods with clients, but often felt largely unable to affect behavior given the influence of socio-demographics and community attitudes on the access to and use of certain methods. For example, one provider described that despite counseling a woman on ‘bleeding’ as a potential side-effect following IUCD insertion alongside as well as reiterating that IUCDs are not permanent methods; there was limited uptake.

*“If you feel any problem then you can remove it after one week. But here, no one is ready to use copper -t…this [IUCD] is more used by educated people.”* (Service provider, Agra).

#### Gender relations and FP access

Despite the feeling of putting choice and decision-making in the hands of clients, as the interviews progressed, many underlying complexities and barriers were highlighted by providers. One of the critical findings to emerge from the qualitative interviews was that some providers acknowledged that they perceived that many of their female clients lacked decision-making power and due to this, the provider inferred that women do not need to be offered information about their FP options.

*“It’s like women have no say in the matter. Mostly they do what their husbands wish to do. Hence women feel that before doing anything they must take their opinion first. Whatever the men desire, happens…”* (Anganwadi worker, Allahabad)

Issues related to gender were common; providers perceived that men’s attitudes were at times a barrier to open communication about family planning. There were two main reasons cited by providers for not having proper counseling sessions with men. Providers often suggested that a large family size renders the family unable to provide proper care for children and providers felt that this argument resulted in a negative attitude towards FP because it threatened the man’s sense of being an adequate financial provider.

*“Some men have false pride that they will manage their family; whatever the family size. They say, I have produced the child and I will take care of it. It’s not your problem.”* (Service provider, Aligarh)

*“I generally talk to women. However I have talked to one or two men. The men I have spoken to say that they do not know anything (about family planning) and they do not want to do anything. I try to explain to them but they do not seem to understand.”* (Service provider, Agra)

The gender of the service provider was also at times a barrier to open communication with men. Almost all female frontline service promoters interviewed in this sample stated that they were uncomfortable talking to men about family planning and that men were also uncomfortable with a female service provider.

#### Perceptions of socioeconomic and normative influence on FP knowledge and access

Another common barrier to client’s use of FP among providers was based on their perceptions of clients’ education and living situation. Almost all providers tended to blame ignorance and illiteracy on the part of uneducated and poor clients as a main reason for lack of adoption of FP.

*“The females who have some education, they tend to think about their family size and understand that if the family is small then they can educate their children, keep them disease-free, and have the resources to raise them well. But there are some women, and in my experience, these are mostly un-educated women, who cannot think of these things and do not space their children or adopt proper family planning methods.”* (Service provider, Agra)

During in-depth interviews, service providers noted that they tended to provide FP-related advice to clients on the basis of clients’ education level, limiting the amount of advice they dispensed to well-educated clients, who were perceived by providers to be already knowledgeable about their options. This is illustrated in the quote, below:

*“If we talk to educated couples, they don’t want children immediately…they take some time… but laymen (less educated clients) want a child right after marriage; they don’t even want to know about family planning. Some of them say it’s God gift…and I try to make them understand that if you have too many children then you can’t give them a good life.”* (Service provider, Agra)

Another barrier to counseling was with newly-wed couples as seen in the quote above. Most providers felt that the social and community norms that put pressure on newly-wed couples to prove their fertility by having a child immediately after marriage were too strong to suggest an alternative. This norm was often internalized by the couple. Thus, most providers stated that advising newly-weds, especially those from poor backgrounds, was often an inefficient use of time and efforts.

*“Newly-wed couples, they think that they should have one or two children first; then think about family planning.”* (Service provider, Agra)

In general, providers spoke of challenges facilitating intra-familial dialogue about FP, dispelling misconceptions, and yet felt that community perspectives (particularly women’s) were beginning to change toward more open acceptance and use of modern FP methods.

## Discussion

In urban Uttar Pradesh, India, where modern FP use is approximately 48% and health facilities tend to provide a reasonable method mix [23], investigation into other restrictions to clients’ access to FP becomes increasingly important. This study of reproductive health service providers in urban Uttar Pradesh shows that providers unnecessarily restrict clients’ access to FP methods, and these restrictions are based on neither government policy nor valid medical justification. The quantitative findings show that providers report restricting clients’ access to FP based on age, parity, partner consent and marital status, while the qualitative findings reinforce that providers, at times, make judgments about their clients’ education, FP needs and ability to understand FP options. These provider-imposed restrictions may mean that women who are young, unmarried or newly married, have few or no children, do not have the support of their partner, or are less educated may not be able to access or use FP or their preferred method. Additionally, providers commonly reported maximum age restrictions for FP meaning that older women may have restricted access to methods, despite potentially still being well within childbearing age.

Our results corroborate findings from other studies in Africa as well as studies in Asia [4,5,14,17,31,32]. For instance, Schuler and colleagues (1985) found that service providers inappropriately restricted access to clients based on education level, parity, caste and wealth in rural Nepal [17]. A study from Pakistan showed that community-based providers also make judgments based on parity and age before supplying FP [32]. Further qualitative research is needed to better understand these types of barriers and to inform programs to train providers on interpersonal communication and provision of methods.

The findings of this study also may be a product of providers adhering to cultural practices that are guided by strong patriarchal norms that are common in Uttar Pradesh, India [33]. These norms lead to gender inequality in UP, resulting in women having a less empowered role in society. As the qualitative findings demonstrate, providers may perceive that women have little power to independently make choices about FP use or their own fertility; therefore providers may decide what methods to offer based on factors such as age, marital status, parity, and education. These providers may combine this information with existing cultural norms as guidance for FP method provision, even if not medically appropriate.

One finding of particular importance is that providers restrict access to spacing methods among lower parity women who may be in need of spacing rather than limiting methods. For example, 20% of doctors and 65% of nurses impose a parity requirement for use of pills, a common spacing method that is medically acceptable to use at any parity. This may be due in part to how government policies have shaped FP method use and method availability. Traditionally, government policies in India emphasized the use of female sterilization and neglected temporary methods [34,35]. As a result, the most commonly used FP method in India is female sterilization, and nearly three quarters of women in urban India report having never used FP prior to the birth of their second child [19,35]. Increasing uptake of pills, and other spacing methods in general, requires that providers offer spacing methods to all women who want to delay a pregnancy. Alternatively, providers may be guiding FP provision for their clients based on the clients’ family size and composition, which may manifest in providers inappropriately deterring women from using both spacing and limiting methods until they have sons.

This study also shows that pharmacy workers tend to have fewer reported restrictions for method provision as compared to health facility-based providers. Being that many men and women in India obtain their condoms and pills from pharmacies [23], access to family planning may be less of a problem for these temporary methods than the long acting methods that must be obtained from a health facility. Only about 28% of pharmacy providers reported having ever received any training on FP, so even though they may not frequently restrict access to methods based on eligibility barriers, they may not have the knowledge and training to appropriately counsel and provide the methods.

Additionally, facility-based providers also report low levels of having ever received training on FP, and among those that had ever received FP training, one quarter or less received it in the last one year. Training on FP could equip providers with the knowledge of when and how to provide FP methods to clients, therefore resulting in reduced barriers to method provision. In the case of methods with lower restrictions due to eligibility barriers, such as condoms, these providers could benefit from training on appropriate counseling and provision of methods.

The pattern that emerges from the qualitative findings is that a particular profile of clients-under-educated, poor, and newly-wed women - are less likely to receive FP counseling by a provider in the urban Uttar Pradesh sites studied. These findings point to the need to develop training programs for providers-these programs should frame family planning in terms of universal human rights. Moreover, although provider training did not seem to emerge in the qualitative data, given the quantitative findings, there is a need for further qualitative exploration around how enhancing provider support and continued education affects FP access in urban Uttar Pradesh.

Providers that are from the same communities in which they work may be more suited and more successful in providing FP to clients in that community [8]. Community-based workers, such as service promoters, may better understand the needs of people in their community and their views likely reflect social norms and mores of this community. Efforts should be made to locally recruit service providers from all cadres in order to minimize social disconnects between clients and providers.

Our results also show that providers report generally following Government of India guidelines for female sterilization, though some doctors report requiring women to have two or more children before undergoing sterilization where the guidelines require at least one child [25]. Doctor’s response to GoI sterilization guidelines may be interpreted in different ways. It may be that guidelines are new and not all providers have been trained to date on these guidelines. Alternatively, it may be that some doctors still impose barriers because they are uncomfortable with the parity guidance. Further qualitative research is needed to better understand the roll-out of the GoI guidelines toward sterilization.

This study has a number of limitations worth noting. Though this survey included a census of high volume facilities and public facilities, it neither had a census of private clinics/doctors and pharmacies nor a random sample of them. Included private facilities were based on preferred facilities and pharmacies reported in the individual survey, therefore the facilities are not necessarily representative of the city as a whole. Given the large number of private providers in the study cities and a lack of a systematic list of these providers, our approach ensures that we survey the facilities and providers where women in our study sample actually said they went for reproductive health services, thus permitting linkages between the household and facility-level surveys. This study also did not collect any information on the background characteristics of the providers; this information could have been useful in further analysis. Finally, another limitation is that this study was only carried out using data from providers. Though client exit interview data was collected for the MLE study, there is no way to link the client exit interview data to the provider that they saw, and therefore no way to corroborate the information that the provider gave. This is a potential source of bias, as providers may respond with what they believe is the medically appropriate response, rather than what they do in reality.

Despite these limitations, this study is unique in that it uses mixed methodology research to explore socially and culturally imposed restrictions on method provision. To the authors’ knowledge, this is the first study of its kind to be carried out in urban Uttar Pradesh. Investigation into provider barriers in the Indian context may help to further elucidate reasons why women may not be using FP or using the method they prefer.

## Conclusion

With poor health indicators, Uttar Pradesh is a key state in which to focus government efforts at improving health delivery services. Ensuring that health facilities have the appropriate guidelines and other materials related to method provision could help reduce inappropriate restrictions to method use. Furthermore, in-service training for all staff is needed, with a focus on reviewing the current guidelines and eligibility criteria for provision of family planning methods. Providers as well as pharmacy staff can also benefit from training that sensitizes them to treating clients, particularly women, with more respect and incorporating a less hierarchical environment during consultations. This type of training should also extend to medical students and future doctors to dispel these barriers during pre-service training. This training can reduce provider-imposed restrictions to use and increase the likelihood that women are able to adopt family planning when they want to delay or limit childbearing in urban India. These types of training programs should lead to reductions in provider imposed barriers to FP and improvements in the quality of FP services for all.

### Endnotes

^a^An Ayush provider is one that practices Ayurveda, Yoga & Naturopathy, Unani, Siddha and Homoeopathy and is certified through the Department of Ayurveda, Yoga & Naturopathy, Unani, Siddha and Homoeopathy (AYUSH). ^b^Traditional birth attendants in India may be either trained or untrained TBAs. ^c^An Anganwadi worker is a community based, voluntary frontline worker who is from the same community in which she works. ^d^A service promoter is a frontline health worker, or community health worker, who was working to provide information about FP and promote FP services to women and households in the UHI project areas.

## Competing interests

The authors declare that they have no competing interests.

## Authors’ contributions

All authors contributed to the conception and design of the study. LMC and ISS conceptualized the study. LMC performed the quantitative analysis and drafted the manuscript. RR, PS, and NC undertook the qualitative analyses. All authors interpreted the data and participated in revision of the manuscript. All authors read and gave final approval for the version submitted for publication.

## Pre-publication history

The pre-publication history for this paper can be accessed here:

http://www.biomedcentral.com/1472-6963/13/532/prepub
